# Polymeric Microneedle Drug Delivery Systems: Mechanisms of Treatment, Material Properties, and Clinical Applications—A Comprehensive Review

**DOI:** 10.3390/polym16182568

**Published:** 2024-09-11

**Authors:** Yun Liu, Ruiyue Mao, Shijia Han, Zhi Yu, Bin Xu, Tiancheng Xu

**Affiliations:** Key Laboratory of Acupuncture and Medicine Research of Ministry of Education, Nanjing University of Chinese Medicine, Nanjing 210023, China; 20200413@njucm.edu.cn (Y.L.); 18149710615@163.com (R.M.); 13058659482@163.com (S.H.); yuzhi@njucm.edu.cn (Z.Y.)

**Keywords:** polymers, microneedle drug delivery systems, drug specificity, biocompatibility, mechanical strength

## Abstract

Our comprehensive review plunges into the cutting-edge advancements of polymeric microneedle drug delivery systems, underscoring their transformative potential in the realm of transdermal drug administration. Our scrutiny centers on the substrate materials pivotal for microneedle construction and the core properties that dictate their efficacy. We delve into the distinctive interplay between microneedles and dermal layers, underscoring the mechanisms by which this synergy enhances drug absorption and precision targeting. Moreover, we examine the acupoint–target organ–ganglion nexus, an innovative strategy that steers drug concentration to specific targets, offering a paradigm for precision medicine. A thorough analysis of the clinical applications of polymeric microneedle systems is presented, highlighting their adaptability and impact across a spectrum of therapeutic domains. This review also accentuates the systems’ promise to bolster patient compliance, attributed to their minimally invasive and painless mode of drug delivery. We present forward-looking strategies aimed at optimizing stimulation sites to amplify therapeutic benefits. The anticipation is set for the introduction of superior biocompatible materials with advanced mechanical properties, customizing microneedles to cater to specialized clinical demands. In parallel, we deliberate on safety strategies aimed at boosting drug loading capacities and solidifying the efficacy of microneedle-based therapeutics. In summation, this review accentuates the pivotal role of polymeric microneedle technology in contemporary healthcare, charting a course for future investigative endeavors and developmental strides within this burgeoning field.

## 1. Introduction

A microneedle drug delivery system can effectively make up for the disadvantages of uncontrolled absorption, reduced risk of drug metabolism, slower drug delivery, and poor patient compliance with the traditional drug delivery modes [1,2]. The traditional modes of administration are intravenous, intramuscular, oral, etc., which are selected according to the needs of the application scenario. The advantages of various drug delivery strategies vary, but the disadvantages are mainly focused on the following aspects: the reduction of the effective concentration of the drug by the hepatic first-pass effect and the alteration of the solubility of the drug by gastric juices and enzymes in the gastrointestinal tract can lead to a decrease in the bioavailability of the drug. Another example is that intramuscular injections are safe but have limited speed and efficiency of delivery, and intravenous injections are fast-acting but have a narrow range of drugs. Therefore, exploring drug delivery routes with high drug absorption and high bioavailability can promote drug development and modernization and enable individualized precision therapy. Microneedle drug delivery systems are typically fabricated from substances such as water-soluble carbohydrates or polymers, which encapsulate the medication entirely within a microarray configuration [3]. This encapsulation property allows the drug to be rapidly dissolved or degraded into the body, with high bioavailability. The polymer materials, characterized by their high immunogenicity and stability, efficiently encapsulate and present antigens, thereby ensuring the drug’s safety profile [4]. Therefore, microneedle drug delivery systems provide an effective solution for rapid drug onset of action through their unique delivery mechanism and characteristics.

Transdermal drug delivery systems are widely used due to their relatively controllable dosage, low toxicity and side effects, and high patient compliance. Since the preparation of nitroglycerin ointment, transdermal drug delivery techniques have consistently pursued controlled or prolonged drug delivery to improve dosage efficacy for precise dosing of therapy [5]. The permeability of different drugs in the skin varies and is influenced by a number of factors, such as the size of the drug molecule, polarity, and skin condition [6]. The main obstacle to transdermal drug delivery systems is the stratum corneum, as it allows only certain molecules (e.g., lipid-soluble substances and nonionic drugs) to passively penetrate the skin [7]. Therefore, chemical enhancements such as penetration enhancers and physical enhancements such as ultrasound input therapy and iontophoresis are currently a hot topic in the exploration of transdermal drug delivery systems to improve efficacy, as is the exploration of novel formulation science methods [8].

Drugs delivered through the skin can directly participate in the systemic circulation and can be locally targeted to accumulate in subcutaneous tissues, such as muscle, bone, and so on [9]. Therefore, the choice of the site of stimulation (whether there is a specific association of the skin localization with the target) for transdermal drug delivery systems is particularly critical. The water–acupuncture technique, a traditional means of acupuncture treatment, can be viewed as a more primitive drug delivery acupuncture technique. Hydro-acupuncture therapy is a therapy guided by the basic theories of traditional Chinese medicine and combines the pharmacological effects of drugs and injection methods [10]. Through acupuncture needles, drugs can be injected directly into acupuncture points, using the dual action of acupuncture and drugs to produce synergistic effects and improve therapeutic efficacy, but there is still room for optimization. Advancements in material science have significantly enhanced the needle–drug conjugation technology. This progress is aimed at bolstering the stability and efficacy of drug delivery by integrating a sophisticated delivery system. The selection of suitable drug carriers is paramount, and the conjugation of drug molecules with these carriers is achieved through a combination of physical adsorption and covalent bonding techniques [11]. This approach ensures a more targeted and controlled release of therapeutic agents. Xu et al. designed the surface of stainless-steel material at the needle tip for the delivery of various payload molecules, wherein the sugar ring allows the encapsulation of structurally determined single or multiple payload molecules by complexation [12]. The above-mentioned delivery system ensures that the drug could be delivered precisely in the joint capsule below the injection site, slowing down the progression of osteoarthritis in the knee joint biochemically and histologically. Advancements in material science have significantly augmented the innovation of acupuncture methodologies. The once rudimentary composite of the water–acupuncture technique has been transformed, with the needle surface now adorned with drug molecules. This upgrade not only enhances its drug-carrying capacity but also paves the way for the targeted and efficient treatment of compromised tissues through acupuncture, marking a significant stride in the evolution of needle-based therapeutics. Advancements in needle technology have also improved the types of stimulation used in body surface medicine and guaranteed patient compliance. Nonetheless, challenges pertaining to drug loading, release kinetics, and the stability of the formulation must be rigorously addressed [13,14,15]. Concurrently, the long-term safety and biocompatibility of the polymer materials within a biological context warrant further exploration and substantiation. There are numerous reviews on microneedles; however, as scholars in the field of Traditional Chinese Medicine (TCM) acupuncture, we place a greater emphasis on the potential effects brought about by the specificity of microneedle stimulation sites. This focus provides fundamental insights into the observation of various acupuncture effects. Such consideration of site specificity is not necessarily shared by most researchers in engineering or Western medicine microneedle studies, and this serves as one of the key motivations for writing this article.

This study provides a comprehensive overview of the microneedle drug delivery system, encompassing its production methodology, operational mechanisms, and preliminary outcomes from animal studies. We advocate that future endeavors should focus on optimizing the design and fabrication of polymer microneedles to enhance their therapeutic potential and ensure their efficacy in clinical applications. The use of various microneedle delivery systems has accelerated the scientific progress of drug formulations, including traditional Chinese medicine, in new forms. The quantitative pharmacological research and device development have led to the integration of diverse therapeutic approaches through modern engineering technologies [16]. Ultimately, we want to increase patient comfort and the system’s therapeutic effectiveness while also doing more to further the modernization and globalization of Chinese medicine.

## 2. Polymeric Microneedle Drug Delivery Systems

Microneedles are typically characterized as slender needles, measuring 10–2000 μm in length and 10–50 μm in width. When meticulously arranged into arrays, these microneedles significantly augment the efficacy of transdermal drug delivery, ensuring the precise and timely application of therapeutic agents [17]. The mechanical properties of polymer microneedles are determined by material and geometrical aspects (shape, aspect ratio, tip radius and length, basal alignment, etc.) [18], which affect their depth of penetration [19], skin insertion ability and fracture force [20]. It has been shown that pyramid-shaped cones have a larger cross-sectional area for the same base width, and thus are mechanically stronger [21]. Ideal polymeric microneedles should have the following characteristics: (1) biocompatibility without triggering an immune response; (2) sufficient mechanical strength to penetrate the stratum corneum; (3) the ability to avoid damage during the manufacture of sensitive cargoes (e.g., proteins, peptides, and vaccines); and (4) controlled delivery of drug release in the skin.

### 2.1. Substrate Materials for Polymeric Microneedle Preparation

Polymer microneedle systems are rich in substrate materials, and the performance of different materials may be affected by factors such as preparation process, additive type, and content. In general, polymer microneedle substrate materials can be divided into three main categories: soluble, insoluble, and hybrid microneedles. The materials used to prepare soluble microneedles mainly include two categories: soluble and degradable. The former mainly includes hyaluronic acid (HA), polyvinylpyrrolidone (PVP), and poly-L glutamic acid (γ-PGA) [14] (Table 1). These materials have good intradermal dissolution performance and short residual time of the needle body in the body, so they have good drug loading capacity and biological safety [22]. Polymer microneedles can be optimized by adjusting the composition of the material. Chen et al. [23] formed microneedle arrays with complementary mechanical properties by adding poly (p-dioxocyclohexanone) (PPDO) as a dispersed phase and blending it with a PLA matrix, which effectively addressed the shortcomings of pure PLA microneedle arrays, such as the tendency of the tip to easily fracture and poor skin adhesion. The biodegradability of polylactic acid-hydroxyacetic acid copolymers (PLGA) can be controlled by adjusting the ratio of lactic acid and hydroxyacetic acid [24,25]. Polymer microneedles can be further improved by adjusting the preparation process (e.g., mold design, molding conditions, etc.) to enhance microneedle performance. Vacuum conditions are formed inside the mold cavity to reduce gas resistance and promote the removal of gas bubbles from the solution [26].

The microneedle–skin interface allows the drug to reach the specified depth of the skin and enter the subcutaneous capillary network to be absorbed, facilitating drug penetration without causing pain or skin damage. Therefore, the mechanical angle of the microneedle is one of the key factors in determining the effectiveness of the microneedle system. The choice of material (e.g., metal, silicon, biodegradable polymers, etc.) and the hardness of the microneedle have a significant impact on its penetration and the degree of skin damage. Harder materials, such as polyether ether ketone (PEEK), as a semi-crystalline polyaromatic crystalline thermoplastic polymer with high hardness and mechanical strength, are suitable for use in the production of polymer microneedles that require a certain degree of hardness and abrasion resistance [27], but may also increase the risk of skin damage [28]. Therefore, the penetration of polymer microneedles is closely related to their shape, size, and material properties, which need to be balanced in order to select the most suitable material for a particular application.

**Table 1:** Comparison of physical properties of major materials for polymer microneedle drug delivery systems.

Material	Hardness	Modulus of Elasticity (Gpa)	Tensile Strength (MPa)	Elongation at Break (%)	Stability	Dissolution Rate	Biocompatibility	Used Routes
PLGA	Strong enough to penetrate skin	1–3	50–100	50–200	At temperatures above 100 °C, it converts to the glassy phase [29]	Slow degradation over days to months [30]	Compatible with PMM, CD	Preparation of degradable microneedles for drug release [31]
PVP	Strong enough to penetrate skin, blended with other polymers to optimize performance, e.g., copolymer PVP-MAA with 1% MAA doubles the mechanical strength of pure PVP microneedles; 25% MAA increases the mechanical strength up to four times [18]	0.5–1.5	30–60	100–300	Melts at 50 °C [32]	Dissolves within 1 min of insertion into skin [33]	Compatible with MAA, PVA [18]	Preparation of soluble microneedles for drug delivery [34]
PVA	Blending with PVP improves hardness	1–2	40–80	50–150	Prevents denaturation of encapsulated drugs by heating and freezing [20]	PVA needles are microcrystalline cross-linked, do not dissolve in the dermis, and can be withdrawn intact after release [35]	Weak interaction between PVP and PVA [19]	Enhanced mechanical strength of microneedles as a coating material [36]
PCL	Modification of PCL with gelatin embedding technology increases mechanical strength by up to two times	0.5–2	20–50	50–250	Melting point of 60 °C, glass transition temperature of −60 °C [37]	Microneedle tip remains in the skin 1 h after insertion [14]	PVA/PVP patches are biocompatible with PCL and act as a support array [38]	Preparation of pharmaceutical coatings for non-heat-resistant drugs [39]
HA	Strong enough to penetrate skin	0.1–0.5	10–30	800–1200	Prevents denaturation of encapsulated drugs by heating and freezing [20]	Penetrates into isolated human skin epidermis and dissolves within 10 min of insertion of tip [40]	Forms PEEK/HA composites with PEEK-based filaments, compatible with most materials [41]	Preparation of soluble microneedles for transdermal drug delivery [42]
PLA	High hardness at 39 °C	3–4	40–60	4–10	Aging at 39 °C, high stability performance [43]	The degradation rate is constant at 25 μm/h under alkaline conditions [44]	Compatible with PMM, CD	Preparation of soluble microneedles for drug delivery [43]

The physical parameters in the above tables are relative and do not represent precise quantitative data. Actual data may vary depending on specific preparation, methods, and conditions. PLGA, polylactic acid hydroxyacetic acid copolymer; PVP, polyvinylpyrrolidone; PVA, polyvinyl alcohol; PCL, polyprolactone; HA, sodium hyaluronate; PLA, polylactic acid.

### 2.2. Properties of Polymer Microneedle Systems

Microneedle arrays have been shown to maximize the amount of substances that can be delivered transdermally by disrupting the stratum corneum and providing an effective channel for drug delivery to the epidermis [45]. Only when fully inserted into the skin can the drug carried by the microneedle be dosed into the body, as designed to work. The length of the microneedle varies from 25 μm to 2 mm, and the tip of the needle is typically structured in a three-dimensional array, with either a beveled shape or a symmetrical taper [46]. It can pass right through the stratum corneum of the skin without touching the painful nerves, creating a temporary drug delivery channel on the skin surface. Microneedle penetration through the skin is the greatest obstacle to polymer microneedle [47], and water-soluble polymers are usually lower in mechanical strength compared to non-dissolvable material. Microneedle insertion into the skin consists of three phases, namely deformation (no penetration), steady-state penetration, relaxation and withdrawal. Mechanical properties are the key to influence the microneedle puncture into skin tissues and effective drug release, and the main focus is on the characterization of the skin puncture properties and the mechanical strength of the microneedle body. The tip force acting axially on the tip of the needle, the axial friction force on the axial side wall, and the normal clamping force on the axial side wall combine to form the mechanical process of microneedle puncture into the skin [48]. When microneedles are inserted into pressurized soft tissues, tip shape parameters such as microneedle height, diameter, and aspect ratio can all directly influence tip force and are positively correlated with cutting force [49]. Henry et al. [50] found that silicon microneedles with a height of about 150 μm can increase the rate of drug penetration into the skin by as much as four orders of magnitude, and can be regarded as one of the candidates with better skin penetration power. John et al. [51] found that microneedles with a height of more than 100 μm acted with greatly enhanced skin permeability, allowing more effective local or systemic therapeutic effects of drugs to be achieved. Within a certain range, the larger the micro-needle spacing (e.g., more than 0.4 mm), the smaller the piercing force, to avoid micro-needle fracture damage. As the piercing displacement tends to stabilize, the piercing efficiency increases [52]. Friction is mainly influenced by Coulomb friction and viscous friction. The normal force is the total clamping force determined and its magnitude increases with the contact area between the needle and tissue during needling. Clamping force, in turn, is influenced by the shape of the incision created by the needle tip in the tissue, as well as the needle pitch. Improving the shape of the microneedle (e.g., reducing the diameter and length, optimizing the clamping angle and tip radius of curvature) can effectively optimize the friction and normal force of the polymer microneedle into the skin [53]. The total axial force of the needle is the sum of the tip force and the friction force. Evaluating the piercing force is also one way to reflect the mechanical strength of the microneedle. The piercing force should be less than the microneedle breaking force so as to ensure that the microneedle does not break and fail during use. The force applied to the microneedle should also be designed to be greater than the insertion force to allow the microneedle to penetrate the skin effectively, while controlling the strength of the force to minimize pain for the user.

## 3. Skin Tissue Properties at the Site of Stimulation Mediate Microneedle Action

### 3.1. Transdermal Drug Delivery: Subcutaneous Microcirculatory Oscillations Promote Drug Absorption

Microcirculation exchanges substances through capillary walls and is responsible for functions such as nutrient absorption, excretion of waste and carbon dioxide, immune defense, tissue repair and regeneration, and maintenance of a stable internal environment [54]. The mean blood flow (MBF) at acupoints is greater than that of the surrounding tissues, suggesting that there are differences in the microcirculatory state between the acupoints and the surrounding tissues [55]. When acupoints are stimulated (e.g., needling, patching, etc.), the blood perfusion to the acupoints increases [56]. When drugs are administered by methods such as acupoint injections, they may be more readily absorbed and enter the blood circulation due to oscillations in the microcirculation and increased blood flow in the acupoint area [57]. After being absorbed, the drug will enter the blood circulation, bind with plasma proteins, and then be transported to various tissues and organs to exert its medicinal effects. Subcutaneous microcirculation oscillations in the acupoint area may promote drug absorption by increasing blood flow to the acupoints and changing the frequency of blood flow oscillations.

### 3.2. Microneedle Drug Delivery: Acupoint–Target Organ Ganglion Connection Promotes Drug Target Accumulation

Body surface acupuncture points and internal organs are connected by the ganglionic emission of somatic and visceral nerves [58]. Research on the analgesic mechanism of acupuncture has also proven that this ganglionic connection can more effectively reduce the patient’s pain [59]. Therefore, selecting the corresponding acupuncture points for microneedle drug delivery based on the target organs of the ganglia can improve the efficacy of the drug. Multilayered modified polymer needles with a diclofenac sodium carrier increase the bioavailability of diclofenac sodium at acupoint stimulation, compared to intra-articular injection [60]. The drug delivery interface established between the skin of the acupoint area and the microneedle can be electronically driven at the microneedle–skin interface. The system delivers drugs on demand, synergistically treats rheumatoid arthritis, provides effective analgesia, and extends the use of polymer microneedles [61,62].

## 4. Application of the Polymeric Microneedle Drug Delivery Systems

### 4.1. Diabetes

Insulin administration devices, characterized by prompt in vivo glucose responsiveness, typically exhibit constrained insulin-carrying capacity and present challenges in terms of manufacturing feasibility. Hence, an approach being pursued in microneedle systems for blood glucose management involves replicating the swift in vivo glucose release response observed in pancreatic β-cells, while also prioritizing high biocompatibility to address acute and long-term toxicity concerns. Microneedle patches offer a distinct advantage by circumventing the skin barrier and facilitating the direct delivery of macromolecules through an array of microneedles [63]. This feature enhances their efficacy in minimizing pain, rendering them a preferred option for individuals with chronic ailments such as diabetes, necessitating prolonged therapeutic interventions. Ma and colleagues have developed a novel approach that incorporates dopamine nanoparticles (PDA NPs) within the external layer of a microneedle system [64]. This design enhances the adhesion of the microneedles to skin tissue, thereby facilitating the efficient healing of diabetic wounds. Mesenchymal stem cell (MSC)-derived synthetic nanovesicles (NVs), created through extrusion via a porous membrane [65], demonstrated a notable 250-fold rise in production yield, along with substantial improvements in mRNA and protein expression levels [66]. This potent therapeutic growth factor exhibited by the PDA microneedle patch provides notable advantages in promoting angiogenesis, reducing inflammation, and enhancing collagen regeneration. Potential advantages of polymeric microneedle drug delivery systems in the management of wound healing were also demonstrated [67].

### 4.2. Oncology

The microneedle drug delivery system fully wraps the drug within the micro-array, enabling the drug to enter the stimulation site and the corresponding subcutaneous tissues and to be rapidly dissolved or degraded, with high bioavailability. Microneedling is becoming one of the most effective drug delivery methods for skin cancer and other cancer treatments [68]. Polymer-based micro- and nanocomposites have advantages over traditional metal and glass micro- and nanomaterials, such as eliminating the need for external storage of drugs, providing structural stability, and enabling high drug loading [45]. Polycaprolactone (PCL), which is highly hydrophobic and highly biocompatible, contributing to the improved solubility and bioavailability of low molecular lipid soluble drugs. A hydrophilic crosslinked PVP polymer microneedle system delivers drugs at a more efficient drug release rate [69]. Both PCL and PNVP are highly biodegradable and non-toxic in the concentration range of 500 μg/mL, which improves the efficiency of low molecular weight drug delivery and ensures the safety of long-term use [70]. In addition both of them have good compatibility with blood, which promotes the systemic delivery of drugs. The pharmacokinetic process simulated by the polymer-based hydrogel prepared from carboxymethyl cellulose can be similar to the release process of cellular DOX in vivo, synergizing the treatment of melanoma cancer cells [71]. The long-term durability of polymer-MN insertion sites ensures stable blood pressure and stable pharmacological effects that remain in the body for a long time, effectively improving performance and reducing the risk of infection [72]. In summary, polymer microneedle structures show great potential for improving drug bioavailability, prolonging circulation time, controlling drug release and tumor targeting compared to conventional drug release methods.

### 4.3. Pain

The long-term treatment of chronic pain limits the use of delivery modalities such as parenteral injections to prevent systemic toxicity or infection [73]. The low irritation and good reproducibility of the microneedle drug delivery system effectively reduces treatment resistance caused by patients’ skin nociceptive sensitivity to pain. For secondary pain caused by complex diseases such as diabetes, the microneedle system responds to biophysical cues collected in the subcutaneous tissue for possible physiologic monitoring [26]. Chronic pain-related disorders require the localized specific delivery of therapeutic agents. transdermal delivery systems, such as microneedles, provide a slow-release mechanism for the treatment of chronic pain, ensuring sustained plasma delivery and drug efficacy [74]. The microneedle delivery system significantly reduced frequency of treatment programs for chronic pain management and prevented substance abuse [75]. The therapeutic efficacy of conventional transdermal patches is influenced by the physicochemical properties of the drug (e.g., solubility, partition coefficient, hydrophobicity), loading dose, molecular diffusivity, and polymer layer. Oral meloxicam is effective in improving patients’ osteoarthritis pain, but it is associated with adverse effects, such as ulceration and perforation. Alwan et al. designed a meloxicam–polymer microneedle drug delivery system by varying the scale of polymeric microneedle materials [76]. The system had an axial needle fracture force of 30 N/100 MNs and promoted effective transdermal delivery, with a steady flow and release of meloxicam, with minimal hysteresis. Amodwala et al. designed a microneedle delivery system for meloxicam loading, which penetrates the stratum corneum and then efficiently delivers lorazepam to the targeted site of action [77]. Kochhar et al. utilized photolithography to treat microneedle patches, and drug diffusion through the porous channels left by dissolved drug particles and out of the microneedles significantly increased lidocaine loading capacity [78]. The low irritation and good reproducibility of the microneedle delivery system effectively reduces the resistance to treatment caused by the patient’s skin nociceptive sensitivity to pain. For pain secondary to complex diseases such as diabetes, the microneedle system responds to biophysical cues collected in the subcutaneous tissue for possible physiologic monitoring [26].

### 4.4. Rheumatoid Arthritis

At present, the frontline treatments for rheumatoid arthritis (RA) include Nonsteroidal Anti-inflammatory Drugs (NSAIDs), corticosteroids, and Disease-Modifying Antirheumatic Drugs (DMARDs) such as methotrexate and etanercept. These medications are predominantly delivered through oral administration or via subcutaneous and intramuscular injections. Microneedle arrays for transdermal drug delivery present a promising alternative, potentially circumventing first-pass metabolism and its associated serious side effects, such as hepatotoxicity, myelosuppression, and gastrointestinal bleeding [62]. This approach also minimizes the risk of infection and enhances patient compliance, compared to conventional administration methods [79]. Nanosized natural and synthetic polymers, with diameters less than 200 nm, are chosen as drug delivery carriers. The encapsulation of therapeutic agents within these polymers allows for targeted delivery to inflamed sites, promoting localized drug accumulation and minimizing the overall waste of medication [80]. A recent investigation has demonstrated that the administration of antigens to antigen-presenting cells (APCs) through the use of polymer microneedles facilitates a pivotal interaction with CD4+ T cells specific to the antigen. This interaction effectively modulates the differentiation of T cells into regulatory T cells (Treg), thereby conferring significant anti-inflammatory properties within a living organism [81]. Du G et al. developed hyaluronic acid (HA) microneedles, which are enriched with bee venom peptides and exhibit superior mechanical properties. These microneedles effectively penetrated the skin, delivering the bee venom peptides and consequently suppressing the expression of pro-inflammatory cytokines, notably IL-17. Additionally, they observed an enhancement in the proportion of regulatory CD4+ T cells. This innovative approach holds great promise for the treatment of autoimmune conditions, including rheumatoid arthritis [82]. Yao W et al. have achieved a significant advancement with the development of a novel two-layer microneedle patch for rheumatoid arthritis (RA) treatment. This patch, characterized by its excellent stability and skin penetration capabilities, demonstrates a rapid dissolution rate, disappearing within 10 min post-application. A marked decrease in the inflammatory response has been documented, underscoring its therapeutic potential [83]. Furthermore, microneedle (MN) technology facilitates the delivery of a diverse range of therapeutic agents, including genes, nucleic acids, and antigen-specific modified cells and proteins. The microneedle systems present a highly promising alternative to conventional drug delivery methods for rheumatoid arthritis (RA), offering the potential for cost-effective, safe, and sustained therapeutic interventions.

### 4.5. Vaccine Delivery

Prophylactic vaccination stands as the preeminent strategy for managing infectious diseases, with the mode of administration being pivotal to the success of the intervention. The advent of microneedle system-based vaccine delivery marks a significant advancement, presenting a promising alternative to the traditional methods [84]. A notable challenge in vaccine distribution is the requirement for cryogenic storage and transportation, which restricts accessibility. However, recent studies have demonstrated that microneedle powders (MNP), exemplified by their successful application in BCG vaccine delivery, can be stored at room temperature for over 60 days. This innovation maintains the vaccine’s antigenic properties and immunogenicity, without compromising the integrity or efficacy of the vaccine, thus offering a potential solution to the cold-chain dependency [85]. The BCG vaccine delivered via microneedle powder (BCG-MNP) exhibits equivalent efficacy to the traditional intradermal administration, while notably avoiding skin irritation and the formation of post-vaccination scars [86]. This microneedle-based vaccine strategy efficiently introduces antigens to the dendritic cells (DCs) residing in the skin, thereby inducing a robust local immune response that is not observed with intramuscular injections [87]. The selection of microneedle matrix biomaterials is crucial, as they can modulate the immunogenicity of the vaccine and augment the immune response. Zhao B and colleagues have successfully developed bacteriostatic microneedle patches utilizing a fish gelatin matrix, which significantly mitigates the risk of infection, offering distinct advantages over conventional intradermal injection methods [88]. Furthermore, Gonzalez et al. have showcased the efficacy of microneedle patches for the transdermal delivery of nucleic acids and DNA vaccines. They achieved successful gene therapy and transfection by coating PVA microneedles with small interfering RNA (siRNA) and plasmid DNA [89]. During the COVID-19 pandemic, the utilization of microneedles for vaccine delivery emerged as a viable strategy, eliminating the requirement for professional healthcare administration, and thereby mitigating the risk of virus exposure within healthcare environments [90]. Zosano Pharma has reported that clinical findings indicate the equivalence in efficacy between its microneedle delivery system for a trivalent influenza vaccine and the conventional intramuscular injection method.

## 5. Outlook and Conclusions

### 5.1. Possible Prospective Strategies for Optimizing Stimulation Sites

The drug delivery efficiency of microneedle drug delivery systems is influenced by the thickness of the epithelial cells at the stimulation site and the level of keratinization. Therefore, optimizing the selection of the delivery site is critical for drug efficacy. The oral mucosa is considered to be one of the quickest and easiest sites for drug delivery. The oral mucosa has a rich blood supply, which helps to promote effective drug penetration. In recent years, microneedles have also been often used to deliver drugs across the oral mucosal barrier. The epithelial layers of buccal mucosa and sublingual mucosa of oral mucosa are non-keratinized epithelium with rich blood flow and good permeability, which is a common site for oral mucosal drug delivery. Mannose-polyethylene glycol-cholesterol/lipid A liposome microneedle arrays were developed with pre-Hepatitis B virus surface antigen (ProHMAs) that can be easily inoculated orally by the patient themselves via mucosal inoculation [91]. Compared to injections that only stimulate the immune system, microneedle vaccination stimulates the immune system to fight viral infections. Thus, ProHMAs are expected to be a prophylactic and therapeutic hepatitis B vaccine based on a novel vaccine adjuvant delivery system. Adriamycin nano-needle arrays encapsulated in polylactic acid-hydroxyacetic acid copolymers with an average particle size of 137 nm can diffuse laterally from the insertion point to deep tissues of about 1~2 mm (up to 3 mm) for the effective treatment of oral cavity cancers [92]. Based on the above characteristics of oral mucosa, the oral microneedle drug delivery system is widely used in vaccination [93,94,95], local anesthesia [96,97,98], and insulin delivery [99,100]. Future development will combine the advantages of microneedles and oral mucosa, and more new formulations will certainly be introduced to the market.

### 5.2. Prospects for New Biocompatible Materials with Superior Mechanical Properties

The biocompatibility of polymer microneedle materials is one of the foundations for ensuring drug efficacy. The selection of biocompatible materials is important for the design and application of polymer microneedles, which can improve the performance of microneedles in terms of safety, drug release effect, and flexibility. 5-Aminolevulinic acid (ALA) has a wide range of applications in tumor clearance [101,102]. However, intradermal delivery of ALA in photodynamic therapy for the treatment of subcutaneous diseases is limited due to the hydrophilic and amphoteric properties of ALA. The application of bone marrow mesenchymal stem cells can increase the intradermal effect of ALA [103]. Zhu et al. designed an ALA-loaded sodium hyaluronate microneedle system with higher mechanical strength than ALA that significantly inhibited tumor metastasis and was safe and nontoxic [104]. Photoresponsive polymers are a class of polymeric materials that are capable of undergoing changes in morphology, structure, or chemical properties in response to light [105,106]. They can achieve the precise control of drug release, while utilizing the biological effects of light, such as photodynamic therapy and photothermal therapy, to improve therapeutic effects and reduce side effects [107]. Photoresponsive polymer microneedles can deliver gold nanorods as photosensitizers and drug carriers to the deeper layers of the skin. Excited by near-infrared light, the gold nanorods produce a photothermal effect that kills tumor cells. At the same time, the microneedles can release drug-carrying molecules, such as carcinoembryonic antigen (CEA) and oncoprotein, to activate the body’s immune system as an in situ tumor vaccine [108].

### 5.3. Consideration of Safety Strategies to Increase Microneedle Drug Loading

Skin tissue, which contains a large number of immunologically active cells, severely limits the efficiency of transdermal drug delivery due to its specific physiological structure, further affecting drug bioavailability and efficacy. Currently, polymer needle delivery systems are mainly used to present small molecule drugs, vaccines, and other biologics, and the lack of skin permeability limits the delivery efficiency of large molecule drugs or protein-based drugs. Therefore, decreasing stratum corneum resistance and increasing skin permeability are key issues that need to be addressed in the field of transdermal drug delivery. To improve the permeability of suspended cells, techniques such as ultrasound, ion introduction, electroporation, thermal ablation, and microcrystalline grinding have been developed. However, most of these techniques have not progressed to preclinical testing due to the risk of skin irritation [89]. Osmotic enhancers and precursor drug methods have also been widely explored for use, with limited success [109]. Vora et al. reported a biodegradable polymer [110], soluble dimethylnaphthalene, as a microneedle carrier. The high concentration of the polymer in the gel matrix ensured the tensile strength of the film, resulting in good mechanical strength of such microneedles. It can efficiently carry high and low molecular weight drug active ingredients across the skin and maintain the stability of the drug to prevent degradation and denaturation during storage. Stimulus-responsive polymer microneedles (SR-pMNs) represent an innovative on-demand drug delivery system that leverages physiological changes or external environmental stimuli to achieve a continuous and controlled release of medication. These microneedles provide the uniform and efficacious encapsulation of the active pharmaceuticals, with the added advantage of an increased drug loading capacity [111]. Advancements in patented microneedle technologies have spurred innovation within the field of microneedle fabrication, notably through the incorporation of 3D and 4D printing methodologies [112]. These state-of-the-art techniques have facilitated the expedited and precise production of microneedles, thereby addressing the requirements for large-scale commercial manufacturing. The recent patents exemplifying polymeric microneedle drug delivery systems hold significant importance, encapsulating the cutting-edge advancements in pharmaceutical delivery. These innovations are particularly impactful in enhancing therapeutic efficacy, minimizing adverse effects, bolstering patient adherence, and advancing the field of personalized medicine. A case in point is the microneedle array developed by Desimone et al. [113], as detailed in patent US20180064920A1, which facilitates rapid drug delivery within a brief timeframe and is capable of accommodating multiple active pharmaceutical agents (Table 2). The ingenuity of this approach lies in its ability to meticulously regulate the microneedles’ morphology, dimensions, and drug payload, thereby ensuring a precise and reliable drug delivery mechanism. Concurrently, the safeguard of intellectual property rights through patents has motivated enterprises and research entities to engage in further exploration and investment in microneedle technologies. This, in turn, has hastened the progression of microneedle solutions from the experimental phase to real-world clinical implementations [114]. Polymeric microneedles have demonstrated utility in preclinical studies for the targeted delivery of a diverse array of pharmaceuticals and vaccines [115], encompassing protein-based therapies, insulin formulations, and small molecule drugs [116]. Furthermore, they exhibit promising potential across a spectrum of applications, including cosmetic procedures, gene-editing therapies, and tissue engineering endeavors [117]. Nevertheless, the transition of microneedle technology into clinical practice encounters several hurdles, such as guaranteeing microneedle safety, enhancing patient adherence to treatment [118], and establishing suitable regulatory frameworks and standardized national protocols. Looking forward, with the resolution of these challenges, polymer microneedle systems are poised to make significant contributions across diverse medical domains, including targeted drug delivery, vaccination campaigns, and gene-editing therapies, potentially leading to a transformative era in human healthcare.

## Figures and Tables

**Table 2:** Patent optimization strategies related to polymer microneedle drug delivery systems.

Research Topics	Patent Number	Instrument Description	Application Scenarios/Material Properties
Microneedle and manufacturing method	US2021290829A1 [119]	A microneedle or microneedle device includes a microneedle body, extending from a base to a penetrating tip formed from a silk fibroin-based material, which is easy to fabricate and highly biocompatible. The silk fibroin microneedles can be fully or partially biodegradable and/or bioerodible. The silk fibroin is highly stable, affords room temperature storage, and is implantable. The silk fibroin structure can be modulated to control the rate of active agent delivery.	Since silk fibroin microneedles are prepared under mild conditions, the stabilizing effect of silk fibroin on incorporated active agents, such as proteins, can be combined with the convenience and self-administration of microneedles to produce drug delivery platforms that are safe and easy to self-administer and can be stored at elevated temperatures. The microneedles can carry antibiotics for the treatment of local infections.
	US20180064920A1 [113]	The invention generally relates to microneedle devices, methods of making them, pharmaceutical compositions comprising them, and methods of treating a disease by administering them. Specifically, the disclosed microneedle devices comprise a plurality of biocompatible microneedles having one or more of the following: (i) a curved, discontinuous, undercut, and/or perforated sidewall; (ii) a sidewall comprising a breakable support; and (iii) a cross-section that is non-circular and non-polygonal. Alternatively, the microneedles may be tiered. This article is intended as a scanning tool with the purpose of searching within the particular art and is not intended to limit the scope of the present invention.	Through a sidewall comprising a breakable support and a cross-section that is non-circular and non-polygonal, this kind of microneedle can be used for the delivery of insulin and vaccines, as well as the delivery of various enzymes and growth hormones, and it can be used to treat autoimmune diseases by providing immunomodulators. Microneedling can also be used to diagnose a variety of conditions, such as diabetes, heart attacks, infectious and bacterial infections, or to perform standard blood tests.
	US8414548B2 [120]	The microneedle array device includes a substantially planar substrate having an array of apertures, as well as a plurality of microneedles projecting at an angle from the planar substrate. The microneedles have a base portion integrally connected to the substrate, a tip end portion distal to the base portion, and a body portion in between. Each microneedle has at least one channel extending substantially from the base portion through at least a part of the body portion. The channel is open along at least part of the body portion and is in fluid communication with at least one of the apertures in the substrate.	The microneedles have a basic rectangular cross-sectional shape with a tapered tip and are composed of biocompatible metallic materials. This special structure not only reduces the biological barrier of the skin but also improves the delivery rate of the drug. This structure allows the device to deliver a wider variety of vaccines, as well as other drugs.
	JP7291087B2 [121]	Microneedle arrays are provided to administer medications or other substances into tissue. A method for fabricating an array of microneedles is also provided. The array includes a base substrate, a primary funnel extending from one of the base substrates, two or more medium microneedles extending from the primary funnel portion, and two or more microneedles comprising the desired material. This method provides a non-porous and gas-permeable type, with two or more cavities defining each microneedle; filling the cavity with a fluid material containing the object and liquid vehicle; and removing at least a portion of the liquid vehicle to form multiple microneedles that contain the desired material, including drying the fluid material. The filling is carried out by applying the pressure difference between opposed surfaces of the type.	In microneedle drug delivery embodiments, the substance of interest may be a prophylactic, therapeutic or diagnostic agent useful in medical or veterinary applications; prophylactic or therapeutic substances, referred to herein as APIs, may include representative APIs for administration, such as antibiotics, antivirals, analgesics, antihistamines, anti-inflammatories, anticoagulants, allergens, vitamins, and anti-tumor agents.Microneedle transdermal drug delivery substances include vaccines, such as infectious disease vaccines, cancer treatment vaccines, neurological disease vaccines, allergy vaccines, smoking cessation vaccines, or other addiction vaccines. Examples include anthrax, cervical cancer, dengue, diphtheria, Ebola, hepatitis A, HIV/AIDS, human papillomavirus (HPV).
Microneedle delivery device and method	US9549746B2 [122]	A microprotrusion array for transporting a material across a biological barrier, wherein said array comprises a plurality of microprotusions composed of a swellable polymer composition.	These microneedles, comprising a plurality of microprotusions made from a swellable polymer composition, are used for the delivery of beneficial substances across or into the skin or for the monitoring of levels of substances of diagnostic interest in the body.
	US10953210B2 [123]	A drug delivery device that delivers pharmacologically active substances transdermally using microneedles arranged on a belt mounted rotatably about a plurality of rollers, the microneedles having an associated drug reservoir mounted on the belt that is compressed when the needles and belt are brought into contact with the skin.	The microneedles can consist of various suitable materials, such as silicone, stainless steel, and plastic. The microneedle allows a defined amount of drug to be pumped almost simultaneously through the microneedle holes to a defined depth within the skin. The device ensures that the needle is in good contact with the skin at the point of administration and prevents drug reflux by actively forcing the drug through the microneedle holes into the skin.
	US10716764B2 [124]	The invention concerns a transdermal delivery system for controlled dispensing of an active substance to and through a porous surface. A certain amount of fluid comprising at least one active substance and at least one solvent is dispensed into an administration reservoir. In the administration reservoir at least one solvent is separated from the administration reservoir by a solvent recovery method, allowing the active substance to achieve a certain level on an interface device that is permeable for that active substance. Thus, the active substance is absorbable via diffusion from the interface device through a porous surface to be treated.	Active ingredients which can be administered via the devices of the present invention include pharmaceutical compositions that are capable of transdermal administration. Agents include those which are sufficiently lipophilic or hydrophilic to penetrate the surface of the skin and the stratum corneum. Some of these agents can reach the micro vessels of the skin and are subsequently absorbed and distributed throughout the body. Drugs suitable for transdermal delivery include scopolamine, nitrates such as nitroglycerin, antihypertensive or antiadrenergic drugs such as clonidine, 17-beta-estradiol, and testosterone. In addition to transdermal delivery, the disclosed dispensers can also be used for the topical surface application of drugs such as antibiotics, corticosteroids, minoxidil, or retinoids (e.g., retinol A).
	US20220040466A1 [125]	A medical system. The medical system includes at least one compartment for a fluid, a fluid path, and at least one microneedle fluidly connected to at least one compartment by the fluid path. A medical infusion system is also disclosed. The system includes a wearable housing, at least one non-pressurized compartment for a fluid contained within the housing, at least one fluid path fluidly connected to the at least one compartment, and at least one microneedle fluidly connected to the at least one compartment by the fluid path, wherein the fluid path extends through the microneedle.	This microneedle can be incorporated as a fluid delivery device for administering fluids such as insulin, chemotherapies, vitamins, painkillers, antibacterials, antimicrobials, or any other therapeutic or nutritive fluid or compound therapy.
